# Supplementary material for: The causal role of circulating inflammatory markers in osteoporosis: a bidirectional Mendelian randomized study
Source: Front Immunol. 2024 Jul 18;15:1412298. doi: 10.3389/fimmu.2024.1412298 (PMC11291241; doi:10.3389/fimmu.2024.1412298)
Supplement: Supplementary file 1 [file DataSheet_1.zip › supplementary material/OSTEOPOROSIS-CIM/GCST90274800.tsv.gz_leave_one_out.pdf]

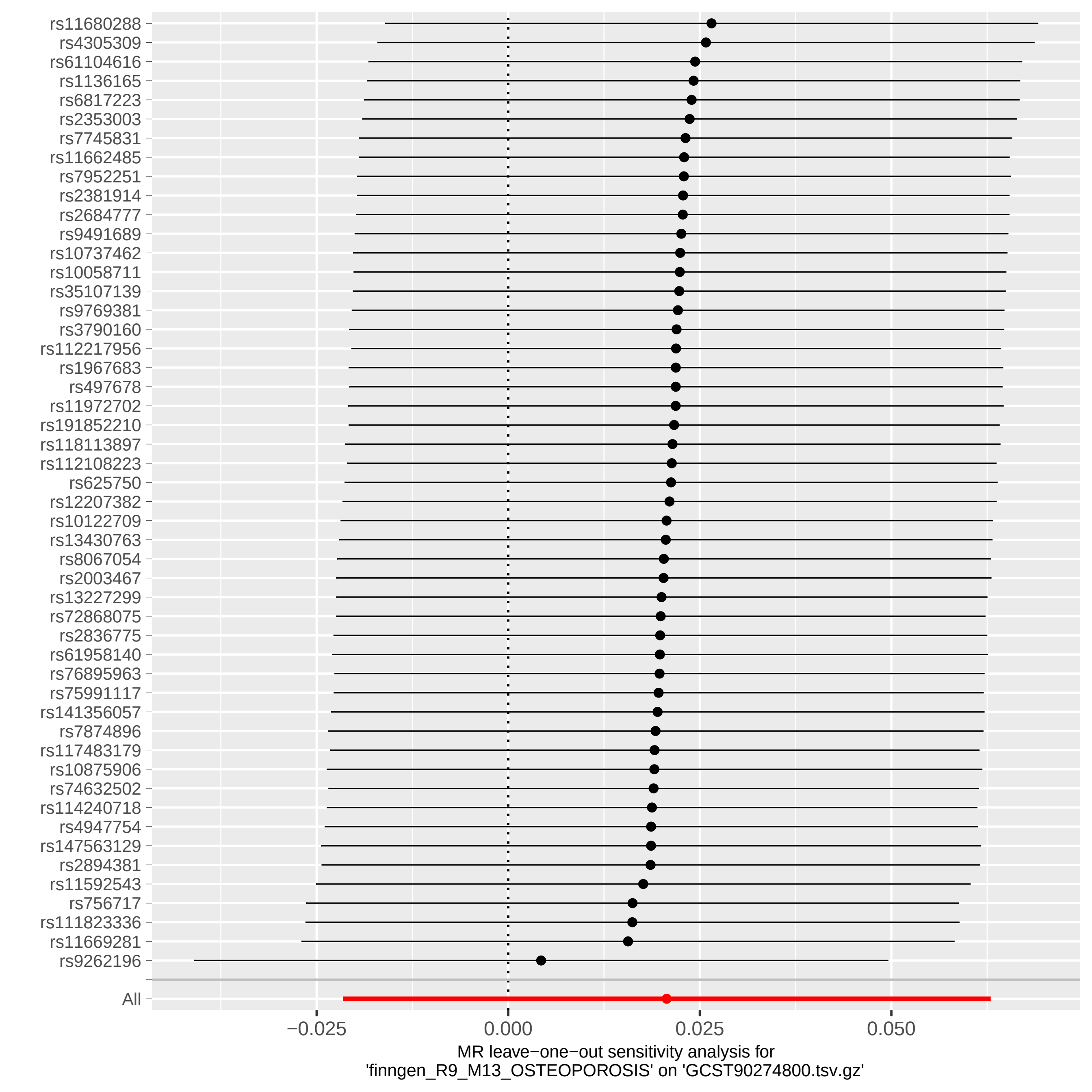
rs11680288
rs4305309
rs61104616
rs1136165
rs6817223
rs2353003
rs7745831
rs11662485
rs7952251
rs2381914
rs2684777
rs9491689
rs10737462
rs10058711
rs35107139
rs9769381
rs3790160
rs112217956
rs1967683
rs497678
rs11972702
rs191852210
rs118113897
rs112108223
rs625750
rs12207382
rs10122709
rs13430763
rs8067054
rs2003467
rs13227299
rs72868075
rs2836775
rs61958140
rs76895963
rs75991117
rs141356057
rs7874896
rs117483179
rs10875906
rs74632502
rs114240718
rs4947754
rs147563129
rs2894381
rs11592543
rs756717
rs111823336
rs11669281
rs9262196
All
−0.025
0.000
0.025
0.050
MR leave−one−out sensitivity analysis for
'finngen_R9_M13_OSTEOPOROSIS' on 'GCST90274800.tsv.gz'
